# Susceptibility of in vitro produced hatched bovine blastocysts to infection with bluetongue virus serotype 8

**DOI:** 10.1186/1297-9716-42-14

**Published:** 2011-01-24

**Authors:** Leen Vandaele, Wendy Wesselingh, Kris De Clercq, Ilse De Leeuw, Herman Favoreel, Ann Van Soom, Hans Nauwynck

**Affiliations:** 1 Department of Reproduction, Obstetrics and Herd Health, Faculty of Veterinary Medicine, Ghent University, Salisburylaan 133, 9820 Merelbeke, Belgium; 2 Department of Virology, Veterinary and Agrochemical Research Centre, Groeselenberg 99, 1180 Brussels, Belgium; 3 Department of Virology, Parasitology and Immunology, Faculty of Veterinary Medicine, Ghent University, Salisburylaan 133, 9820 Merelbeke, Belgium

## Abstract

Bluetongue virus serotype 8 (BTV-8), which caused an epidemic in ruminants in central Western Europe in 2006 and 2007, seems to differ from other bluetongue serotypes in that it can spread transplacentally and has been associated with an increased incidence of abortion and other reproductive problems. For these reasons, and also because BTV-8 is threatening to spread to other parts of the world, there is a need for more information on the consequences of infection during pregnancy. The aim of the present study was to investigate whether hatched (i.e. zona pellucida-free) in vitro produced bovine blastocysts at 8-9 days post insemination are susceptible to BTV-8 and whether such infection induces cell death as indicated by apoptosis. Exposure of hatched in vitro produced bovine blastocysts for 1 h to a medium containing 10^3.8 ^or 10^4.9 ^TCID50 of the virus resulted in active viral replication in between 25 and 100% of the cells at 72 h post exposure. The infected blastocysts also showed growth arrest as evidenced by lower total cell numbers and a significant level of cellular apoptosis. We conclude from this in vitro study that some of the reproductive problems that are reported when cattle herds are infected with BTV-8 may be attributed to direct infection of blastocysts and other early-stage embryos in utero.

## Introduction

Bluetongue virus (BTV) is an orbivirus belonging to the *Reoviridae *family. It has 24 known serotypes and recently a likely 25^th ^serotype has been identified amongst goats in Switzerland [1,2]. Prior to 2006 BTV was known to occur throughout much of the world between latitudes of approximately 40° north and 35° south [3], but a more northerly incursion of BTV serotype 8 (BTV-8) in central-Western European countries was detected which commenced in August 2006. In Belgium, for example, epidemiological studies at the end of 2006 revealed an overall herd and true within-herd prevalence in ruminants of 83.3% and 23.8%, respectively [4]. The economic impact was devastating, with morbidity, mortality, reproductive failure including abortions, transport and export restrictions and other control measures [1,5]. The exact cost of these recent BTV infections has not been calculated, but in 1996, prior to the European outbreaks, worldwide losses due to BTV infections in livestock were estimated to be US$ 3 billion per year [6].

In contrast to infections with other BTV serotypes in other parts of the world, many BTV-8 infected cattle and sheep show clear clinical signs and/or pathological lesions, including fever, crusts/lesions of the nasal and oral mucosa, salivation, conjunctivitis, coronitis, muscle necrosis, and stiffness of the limbs [7]. More importantly, BTV-8 seems to differ from other BTV serotypes in that it spreads transplacentally and thereby may lead to an increased incidence of abortion [8,9]. In the past, transplacental infections with BTV were always thought to have been caused by modified live vaccine strains of the virus [10]. The pathological mechanisms whereby embryos and foetuses are harmed by wild type BTV-8, and the consequences with regards to impaired fertility and/or congenital problems in calves infected in utero remain unknown. Bowen et al. [11] showed that zona pellucida-free bovine and murine morulae are susceptible to infection with BTV-11 and BTV-17 in vitro, with virus replication and cytopathic effects on the embryos. However, studies with BTV-1, -10, -11, -13 and -17 have also shown that zona pellucida-intact bovine embryos could not be infected in vitro [12-14]. Based on these and other studies it was concluded that the risk of transmitting BTV infection during bovine embryo transfer is negligible when the guidelines of the International Embryo Transfer Society (IETS) are followed [15].

The apparently unique ability of BTV-8 amongst BTV serotypes to cross the placenta and to produce prenatal infection indicates that the original research on BTV interaction with bovine embryos should be reappraised [16]. Also, since BTV-8 is threatening to spread more widely over the globe [1], there is an urgent need for more information on the consequences of infection during pregnancy. In vivo derived embryos lose their zonae pellucidae shortly after blastocyst formation and consequently they are likely to be exposed to BTV-8 virus in the uterus during viremia. The aim of the present study was to investigate a) whether hatched (zona pellucida-free) in vitro produced bovine blastocysts (at day 8-9 post insemination) are susceptible to BTV- 8 infection and, if so whether such infection induces apoptosis.

## Materials and methods

### Media and reagents

Tissue culture medium-199 (TCM-199), Basal Medium Eagle (BME) amino acids, Minimal Essential Medium (MEM), non-essential amino acids, and gentamicin were obtained from GIBCO-BRL Life Technologies (Merelbeke, Belgium). For virus exposure Minimal Essential Medium (MEM, 21430-020, GIBCO-BRL Life Technologies) was used. All other media components and reagents were bought from Sigma (Bornem, Belgium). Prior to their use, all the media were passed through a sterile 0.22 μm filter (Millipore Corporation, New Bedford, MA, USA).

### In vitro production of blastocysts

Bovine blastocysts were produced by the in vitro methods used routinely in our laboratory [17]. Briefly, after collecting bovine ovaries from an abattoir, the oocytes were aspirated from follicles measuring between 4 and 8 mm in diameter and cultured for 20-24 h at 38.5°C in 5% CO_2 _in air in groups of 100 in 500 μL modified bicarbonate buffered TCM-199 supplemented with 20% heat-treated foetal calf serum (FCS) (Biochrom AG, Berlin, Germany). Foetal calf serum is a valuable source of growth factors and heat-treatment is used to inactivate any potential microbial contaminants. Spermatozoa were separated out from frozen-thawed bovine semen using Percoll-gradient centrifugation (Pharmacia, Uppsala, Sweden), and then washed. The matured oocytes were incubated with a sperm (sp) concentration of 1 × 10^6 ^sp/mL in an in vitro fertilisation medium consisting of bicarbonate buffered Tyrode albumin lactate pyruvate (TALP) solution, supplemented with bovine serum albumin (BSA, fraction V, A6003, Sigma-Aldrich, Bornem, Belgium) (6 mg/mL) and heparin (25 μg/mL). After 20-24 h of incubation the presumed zygotes were vortexed to remove excess sperm and cumulus cells and subsequently cultured for a further seven days (i.e. to the hatched blastocyst stage) in 50 μL droplets of synthetic oviduct fluid supplemented with amino acids and FCS (SOFaa + 5% FCS) in an atmosphere of 5% CO_2_, 5% O_2 _and 90% N_2 _under mineral oil (Sigma-Aldrich).

### Exposure of hatched blastocysts to BTV-8

We used BTV-8 Bel 2006, an isolate that was originally obtained from an infected sheep by culture of blood on embryonated hens' eggs and then passaged 1 to 3 times on Baby Hamster Kidney (BHK-21) cells [18]. Groups of 4 to 8 hatched blastocysts were exposed to the virus by placing them in 800 μL of MEM containing 10^3.8 ^(experiment 1 and 2) or 10^4.9 ^(experiment 2) tissue culture infectious doses with 50% endpoint (TCID^50^) of BTV-8 and incubating them for 1 h at 39°C in an atmosphere of 5% CO_2 _in air. Mock-exposure of groups of 4 to 8 control blastocysts in 800 μL SOF or 800 μL MEM (two groups) without virus was used to evaluate any negative effects of MEM on blastocyst viability. After 1 h of exposure, all blastocysts in the group were washed ten times by passing through 10 petri-dishes each containing 5 mL of HEPES-buffered TALP, following the guidelines of the IETS manual on embryo processing [15]. The groups of blastocysts were then cultured for a further 48, 60, 72 or 96 h at 39°C in an atmosphere of 5% CO_2_, 5% O_2 _and 90% N_2_.

### Experiment 1: Detection of BTV-8 antigen expression in exposed blastocysts

The proportion of infected blastocysts after each period of culture was determined by indirect immunofluorescence (IF). In order to validate the staining, a BTV-8 infected BHK-21 monolayer was examined in the same way and used as a positive control. All samples were washed three times in polyvinyl pyrrolidone (PVP) solution (1 mg/mL PVP in phosphate buffered saline) and subsequently fixed in 4% paraformaldehyde at 4°C for between 1 h and 12 h. After fixation, samples were stored in a similar PVP solution at 4°C until staining. Prior to staining blastocysts were permeabilised in a detergent (0.5% Triton X-100 (Sigma-Aldrich) in PVP) for 1 h, whereas the positive cell line was permeabilised for just 2 min. In order to prevent aspecific binding of the primary antibody to the samples, blocking was initially done in 10% FCS for 1 h at room temperature (RT). Samples were then washed in a similar PVP solution and incubated overnight in a 1:100 dilution of BTV-8 monoclonal antibody (8A3B.6, ID-Vet, Montpellier, France) at 4°C. Next, the samples were blocked again in 10% goat serum in PVP for 1 h at RT to prevent aspecific binding of the secondary antibody. Afterwards, samples were incubated for 1 hour in a 1:100 dilution of FITC labelled goat-anti-mouse antibodies (Molecular Probes, Invitrogen, Merelbeke, Belgium) and subsequently incubated with 0.5% propidium iodide (Molecular Probes, Invitrogen, Merelbeke, Belgium) for 30 min at RT to stain the nuclei. After mounting the blastocysts, and the positive control BHK-21 monolayer, in glycerol with 1,4-diazabicyclo (2.2.2) octane (25 g/mL), the number of blastocysts positive for the BTV-8 antigen in one or more embryonic cells was evaluated by means of a Leica DM/RBE fluorescence microscope at a magnification of 400× (Leica, Aartselaar, Belgium).

### Experiment 2: Combined detection of BTV-8 antigen expression and apoptosis

The same procedures were followed as in Experiment 1 except that a Texas Red labelled goat-anti-mouse antibody was used as secondary antibody to detect viral antigen expression, and Terminal deoxynucleotidyl transferase (TdT) fluorescein-dUTP Nick End Labelling (TUNEL, Roche Diagnostics, Mannheim, Germany) was used to detect apoptosis. Nuclei were stained with Hoechst 33342 (Molecular Probes, Invitrogen, Merelbeke, Belgium) which selectively binds to double-stranded DNA. Thus, after their incubation in the secondary antibody and two additional washes in PVP, blastocysts were further incubated in the reagent for TUNEL for 1 h at 37°C in the dark, and subsequently washed again in PVP. Finally all of the blastocysts were subjected to Hoechst 33342 staining. Mounting and evaluation were the same as in the first experiment. The apoptotic cell ratio was defined as the percentage of apoptotic cells/total cell number.

### Staining controls

Negatively stained controls were provided by omitting the first, second or both antibodies in the staining procedure and these showed no background staining (resulting data not shown). The degrees of cell cycle arrest and loss of cells in the infected BHK-21 monolayers were increased compared with those in a control, non-infected BHK-21 monolayer. Both staining protocols caused all remaining cells in the infected monolayers to stain positive for BTV-8 (data not shown).

### Statistical analysis

Statistical analysis of the data from experiment 2 for blastocysts examined 48 hours post exposure (hpe) and for those at 72 hpe was done separately. Within each treatment group mixed model univariate analysis of variance was performed to evaluate the total cell number. The apoptotic cell ratio in each treatment group in experiment 2 was also analysed using logistic regression analysis. Being apoptotic or not was included as the dependent variable and treatment was the independent variable.

## Results

### Experiment 1: Detection of viral antigen expression in blastocysts exposed to BTV-8

**Figure 1:**
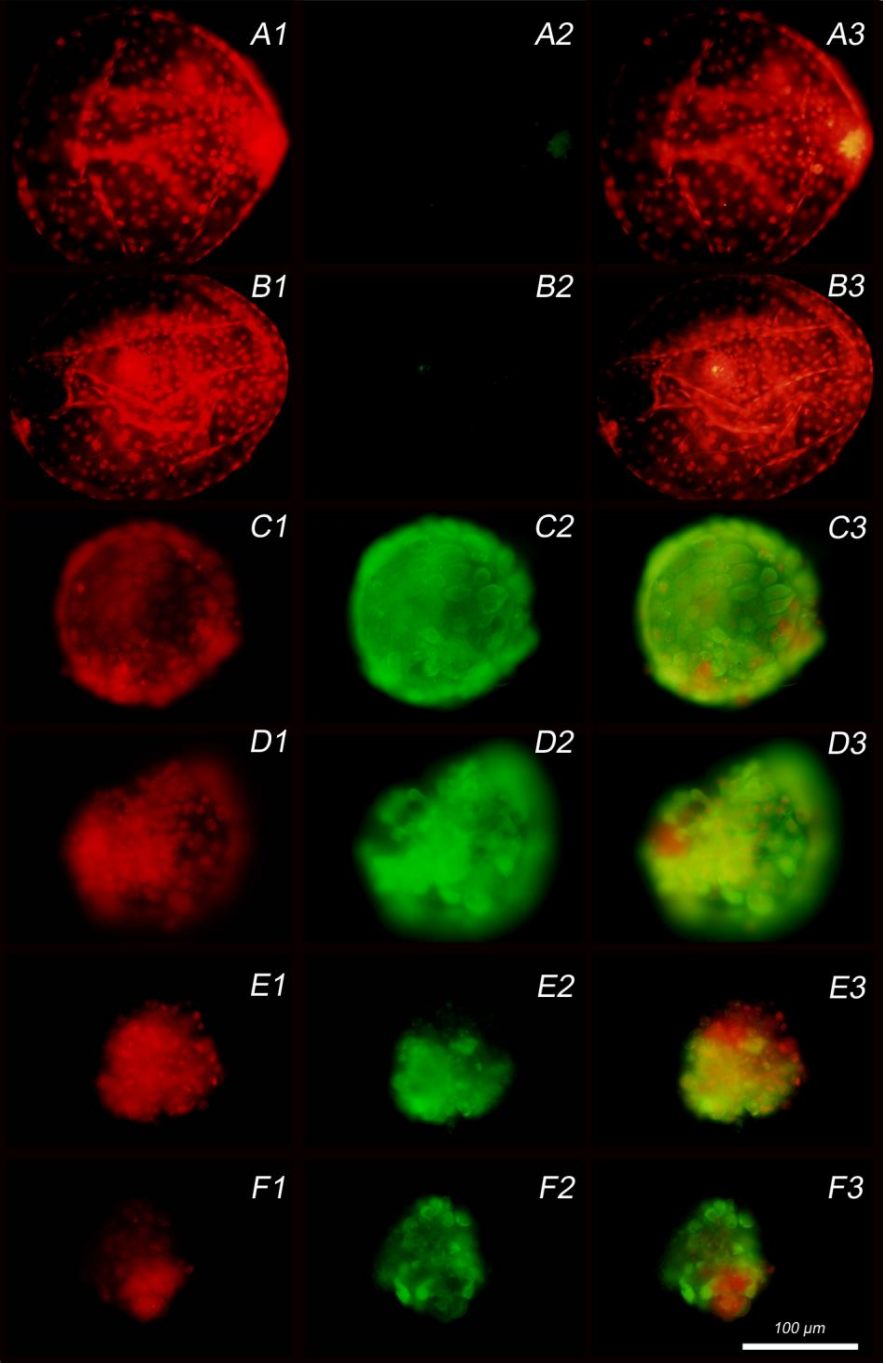
**Blastocysts examined by immunofluorescence microscopy at different time points post exposure to BTV-8**. Column 1: propidium iodide (PI) staining of nuclei; Column 2: indirect fluorescein (FITC) labelled detection of BTV-8 antigen and Column 3: overlay of PI and FITC staining. Row A: blastocyst negative for antigen at 48 h post exposure (hpe). Row B: blastocyst negative for antigen at 60 hpe. Row C: blastocyst positive for antigen at 48 hpe. Row D: blastocyst completely positive for antigen at 72 hpe. Row E: blastocyst positive for antigen at 72 hpe. Row F: blastocyst positive for antigen at 96 hpe.

All of the control, i.e. mock-exposed blastocysts (SOF and MEM) were negative for the BTV-8 virus antigen at all time points (Figure 1, rows A and B). At 48 hpe only one out of 7 exposed blastocysts was positive for viral antigen, but in this case the antigen was detected in all embryonic cells (Figure 1, row C). At 60 hpe, all exposed blastocysts (*n *= 6) were negative, whereas at 72 hpe and 96 hpe all exposed blastocysts had 25% to 100% BTV-8 positive cells (*n *= 6 at 72 hpe and *n *= 7 at 96 hpe) (Figure 1, row D). Furthermore one blastocyst at 72 hpe and 2 blastocysts at 96 hpe showed morphological signs of degeneration (Figure 1, rows E and F).

### Experiment 2: Combined detection of viral antigen expression and apoptosis

**Figure 2:**
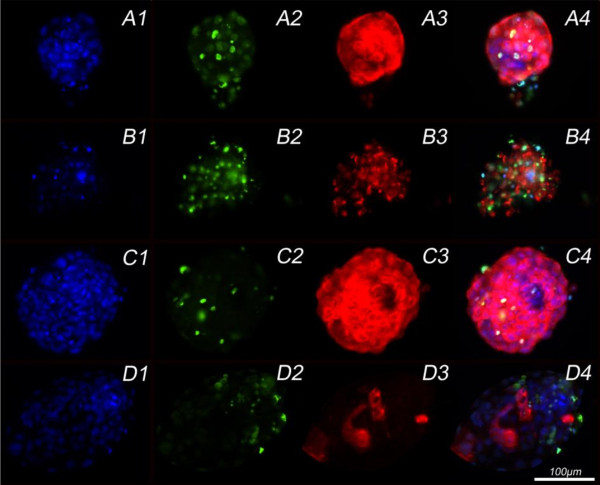
**Blastocysts examined for apoptosis at different time points post-exposure to BTV-8**. Column 1: Hoechst staining of nuclei; Column 2: terminal deoxynucleotidyl transferase (TdT) fluorescein-dUTP Nick End Labelling (TUNEL) for apoptosis detection and Column 3: indirect immunofluorescent staining of viral BTV-8 antigen with a Texas-Red labelled secondary antibody. Column 4: overlays of the three staining procedures. Row A: BTV-8 exposed blastocyst of excellent morphological quality at 48 hpe showing BTV-8 viral antigen in all its cells. Row B: BTV-8 positive blastocyst at 48 hpe showing degeneration and a high proportion of apoptotic cells. Row C: BTV-8 positive blastocyst at 72 hpe with excellent morphological quality. Row D: BTV-8 positive blastocysts at 72 hpe which shows no colocalisation of apoptosis and BTV-8 infection, and which has an excellent morphology.

**Figure 3:**
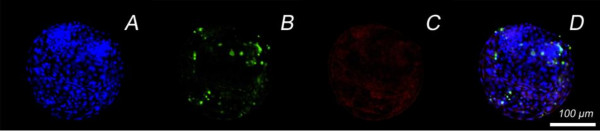
**Mock-exposed, non-infected hatched blastocyst**. A: staining of the nuclei with Hoechst stain; B: apoptosis detection by TUNEL; C: use of Texas Red labelled secondary antibody to show the absence of BTV-8 antigen; and D: a combination of the three procedures.

All BTV-8 exposed blastocysts showed at least some BTV-8 positive blastomeres. At 48 hpe and 72 hpe, respectively, 45% and 38% of the exposed blastocysts were BTV-8 positive in all embryonic cells (Figure 2, rows A and C). Approximately 25% of the blastocysts at both of these time points post exposure showed clear signs of degeneration (Figure 2, row B). The apoptotic cell ratio in the virus exposed blastocysts was significantly higher than in mock-exposed, control blastocysts (Table 1), but when only a few cells were infected, or in cases where there was degeneration, there was no clear co-localisation between the TUNEL (FITC)-positive and the BTV-8 (Texas Red labelled antibody) positive cells (Figure 2, rows B and D). The total cell number was substantially lower in blastocysts exposed to BTV-8 in comparison with mock-exposed, control blastocysts, although this difference was not found to be statistically significant, probably because of the large within group variation (Table 1). Mock-exposed, control blastocysts (SOF and MEM) stained by exactly the same procedure as BTV-8 exposed blastocysts were all negative for BTV-8 (Figure 3).

**Table 1:** Experiment 2: Cell numbers (mean ± SEM) and apoptotic cell ratios (mean ± SEM) of blastocysts in different treatment groups at 48 and 72 hours post exposure (hpe)

Group and treatment	Total cell number (mean ± SEM)	Apoptotic cell ratio (mean ± SEM)
	***at 48 hpe***	***at 48 hpe***

SOF (*n *= 6)	213.3 ± 23.12	14.5 ± 0.98^a^
MEM (*n *= 5)	312.8 ± 100.97	7.0 ± 0.64^b^
BTV 10^3.8 ^(*n *= 7)	107.1 ± 21.47	18.9 ± 1.43^c^
BTV 10^4.9 ^(*n *= 4)	108.4 ± 18.72	11.8 ± 1.55^a^

	***at 72 hpe***	***at 72 hpe***

SOF (*n *= 5)	413.1 ± 119.61	3.9 ± 0.43^a^
MEM (*n *= 7)	331.7 ± 83.24	4.7 ± 0.44^a^
BTV 10^3.8 ^(*n *= 5)	129.3 ± 25.25	14.9 ± 1.29^b^
BTV 10^4.9 ^(*n *= 3)	105.7 ± 23.31	16.6 ± 2.6^b^

Mock-exposed, control blastocysts (SOF and MEM) were incubated in BTV-8 free SOF culture medium and MEM medium respectively. Virus exposed blastocysts were exposed to two different titers of BTV-8 i.e. BTV 10^3.8 ^or BTV 10^4.9 ^TCID50 respectively.

^abc ^Values with different superscript within the same time point and column differ significantly.

## Discussion

During the recent BTV-8 epidemic in central-Western Europe, it was clearly shown that the virus was able to spread transplacentally in between 10% and 33% of the affected pregnant cattle [8,19]. Early transplacental infection with BTV-8 (between 30 and 70 days of gestation) is thought to result in embryonic death and resorption or abortion, which might not be noticed. Later, between days 70 and 130, before the foetus is fully immunocompetent, the infection can affect the foetal central nervous system leading to malformations, such as hydranencephaly [9]. In these cases, the affected calf usually dies within 2 months after birth. Problems such as infertility and diminished health of the offspring have also been demonstrated when infections of the dam occurred after 70 days of gestation [10]. After 130 days, the foetus becomes immunocompent and should be able to clear a BTV-8 infection by production of its own antibodies although the time of complete clearance of the virus or viral RNA will depend on when the intra-uterine infection occurred [8,20].

In the present study, we were able to confirm that infection of hatched blastocysts at the very beginning of gestation can be detrimental to their development. Previous studies with other serotypes of bluetongue virus have shown that the virus can be found in uterine flushings during viraemia, which indicates that embryos are likely to be exposed to the virus, especially after hatching [11]. Our in vitro studies have also shown that after hatching of the blastocyst (around day 8 or 9 after fertilisation) the embryo blastomeres are susceptible to BTV-8 infection, which signifies that the virus can attach to and penetrate through the cell membrane. Attachment and penetration of cells by bluetongue virus is mediated by its two outer capsid structural proteins, VP2 and VP5 [21]. Very recently, cryo-electron microscopy has revealed the structure of these capsid proteins [22]. The protein VP2, which is also called the host attachment protein, has two putative binding sites: an exposed receptor-binding tip and an internal sialic acid-binding pocket. The virus initially binds to the membrane receptor of the host cell with the tip of the VP2 protein, and this is followed by binding via the sialic acid-pocket, which probably stabilises the initial viral attachment [22]. The initial attachment via the VP2 protein is followed by a clathrin-dependent endocytosis and subsequent degradation of VP2 in the endosome of the host cell [21]. VP5, the second outer caspid protein of BTV, is responsible for viral penetration through the cell membrane [22]. Although the exact nature of the receptor for the viral VP2 on the cell is unknown, successful infection of our blastocysts with BTV-8 indicates that these unknown (glyco)protein receptors are present at the surface of the blastomeres of hatched bovine blastocysts. Glycoprotein receptors play important roles during fertilisation, embryo development and embryo implantation [23-25]. Identification of the nature of the VP2 receptor on the cell would allow more detailed investigation of the interactions between BTV-8 and blastocysts and also viral interaction with zona pellucid-intact (unhatched) embryos both in vivo and in vitro.

Once the virus has entered and replicated within the cell, it then needs to leave the cell. In somatic cell cultures the viral VP2 interacts with vimentin which enables egress of virus particles from the cell [26]. The expression of vimentin has been studied in different types of bovine embryos and found to be significantly reduced in cloned embryos, which suggests that these are of lower quality than embryos produced by conventional in vitro procedures [27]. Vimentin has, furthermore, been shown to play a crucial role in normal posthatching development of bovine embryos [28], so it can be assumed that its presence would have enabled the BTV-8 virus to spread within and possibly between the blastocysts in our experiments.

Apoptosis is known to play a central role in the pathogenesis of bluetongue virus infection when the latter leads to tissue injury [29]. Our results showing that BTV-8 infection of hatched blastocysts caused increased apoptosis and developmental arrest suggest that this could have an important role in the decreased fertility that is sometimes observed during BTV-8 epidemics. Mortola et al. have shown that when cells are treated with purified VP2 and VP5 either alone or together, both of these proteins are necessary to induce apoptosis [29]. For reoviruses, however, it has been demonstrated that in addition to the binding of outer viral capsid proteins to the cell receptor, intracellular virus disassembly is also necessary for apoptosis induction, but virus replication within the cell is not essential [30]. This might explain why in some of our BTV-8 exposed blastocysts, apoptosis and virus infection were not colocalised within the blastomeres (Figure 2 row D column 4). When virus spreads within the blastocyst and apoptosis leads to embryonic arrest and death in vivo, the clinical outcome will depend on the timing of embryonic death. Loss of the embryos before day 14 will usually be followed by regular return to oestrus at 21 days whereas embryonic death after day 14 will often result in a delayed return to oestrus.

Having shown in our present work that hatched bovine blastocysts are susceptible to BTV-8 infection, we believe that future research should investigate the effect of insemination with BTV-8 infected semen and also the effect of viremia of the dam during development of early (i.e. zona pellucida-intact) embryos as well as hatched blastocysts in vivo. Since the properties of the zona pellucida are very different between in vitro produced and in vivo derived embryos [16], additional research is also needed to ascertain the susceptibility of both types of zona pellucida-intact embryos to BTV-8 infection. Although it is unlikely that the virus would penetrate through the zona pellucida, the possibility cannot be excluded that virus particles may stick to the zona and might not be removed by the thorough washing procedures advocated by the IETS [15,16].

In conclusion, we have shown that hatched bovine blastocysts are susceptible to infection with BTV-8 and that infection with this BTV serotype results in their retarded development. Total numbers of cells in BTV-8 infected blastocysts were lower than in mock-exposed, control blastocysts, and there was significantly more apoptosis. Consequently, we believe that the reported problems with herd fertility during a BTV-8 epidemic may not be exclusively caused by BTV-8 induced maternal effects in the cows (such as fever), but potentially may also be attributed to direct infection of early embryos in utero.

## Competing interests

The authors declare that they have no competing interests.

## Authors' contributions

LV carried out both experiments and drafted the manuscript. WW participated in the first experiment. KDC provided the BTV-8 BEL 2006 virus and participated in the design of the study. IDL provided the BTV-8 infected cell lines. HF participated in imaging the fluorescent stained samples. AVS participated in the design of the study and in writing the manuscript. HN participated in the design of the study and in writing the manuscript. All authors read and approved the final manuscript.
